# Ginsenoside Rg1 can restore hematopoietic function by inhibiting Bax translocation-mediated mitochondrial apoptosis in aplastic anemia

**DOI:** 10.1038/s41598-021-91471-1

**Published:** 2021-06-17

**Authors:** Huiqin Cao, Wei Wei, Ruirong Xu, Xing Cui

**Affiliations:** 1 Department of Hematology, Affiliated Hospital of Yan’an University, Yanan, 716000 China; 2 Department of Neurosurgery, Affiliated Hospital of Yan’an University, Yanan, 716000 China; 3 Department of Hematology, Affiliated Hospital of Shandong University of Traditional Chinese Medicine, Jinan, 250014 China

**Keywords:** Molecular biology, Medical research, Molecular medicine

## Abstract

The present study investigated, the anti-apoptotic activity of *Ginsenoside Rg1* (Rg1) via inhibition of Bax translocation and the subsequent recovery of hematopoietic function. Mitochondrial apoptosis in bone marrow mononuclear cells (BMNCs) was observed in aplastic anemia (AA) patients. To establish a mouse model of AA, BALB/c mice were transplanted with lymph node cells from DBA/2 donor mice via vein injection after treatment with Co60 γ-radiation. After treatment with Rg1 for 14 days, the peripheral blood and Lin–Sca-1 + c-Kit + (LSK) cell counts of the treated group were increased compared with those of the untreated model mice. In in vivo and in vitro tests of LSKs, Rg1 was found to increase mitochondrial number and the ratio of Bcl-2/Bax and to decrease damage to the mitochondrial inner and outer membranes, the mitochondrial Bax level and the protein levels of mitochondrial apoptosis-related proteins AIF and Cyt-C by decreasing the ROS level. Rg1 also improved the concentration–time curve of MAO and COX and levels of ATP, ADP and AMP in an in vitro test. In addition, high levels of Bax mitochondrial translocation could be corrected by Rg1 treatment. Levels of markers of mitochondrial apoptosis in the Rg1-treated group were significantly better than those in the AA model group, implying that Rg1 might improve hematopoietic stem cells and thereby restore hematopoietic function in AA by suppressing the mitochondrial apoptosis mediated by Bax translocation.

## Introduction

Aplastic anemia (AA) is considered a bone marrow failure syndrome with peripheral pancytopenia and marrow hypoplasia. Exposure to chemicals, drugs, radiation, radioactive materials, and radiation-producing devices, infection, immune disease, heredity (in 50% of cases) and unknown etiologies can lead to the development of AA. Current treatments focus on using immunosuppressive treatment (IST) or replacing hematopoietic stem cells (HSCs) via transplantation (HST). However, there are problems with these treatments, such as the limited efficacy of IST and the serious side effects of HST, such as graft versus host disease. Therefore, treatments aimed at restoring hematopoietic stem cell number and function a represent potential alternative approaches.

Mitochondrial function is essential for cells, including HSCs, because mitochondria are the major site of adenosine-5′-triphosphate (ATP) production^1^. In addition to playing fundamental roles in energy production and metabolism, mitochondria exhibit other important functions, including the maintenance of calcium homeostasis, and regulation of cellular and intracellular signaling, inflammation, and apoptosis^2,3^. The multilineage differentiation and proliferation capacities of HSC are particularly vulnerable to inflammation and apoptosis^4,5^. Bax is a strong multidomain proapoptotic protein that resides in the cytoplasm as an inactive monomer in healthy cells^6^. Upon encountering apoptotic stimuli, Bax undergoes conformational activation, leading to its translocation to mitochondria. Studies have indicated that Bax translocation is a key mechanism of the apoptosis of human monocytes^7^.

*Ginsenoside Rg1 *(*Rg1*) is a steroidal saponin that is highly abundant in ginseng and one of its most important components^8^. Studies have shown that *Rg1* can protect hematopoietic stem/progenitor cells (HSPCs) by attenuating oxidative stress^9^, improving the hematopoietic microenvironment^10^, protecting against X-ray irradiation-induced aging^11^ or protecting against cyclophosphamide-induced myelosuppression in mice by recovering hematopoietic function^12^. Rg1 can alleviate oxidative stress and inflammation^13^, inhibit the excessive activation of Wnt/β-catenin signaling in aged HSPCs^9^, Rg1 protect mitochondrial function by inhibiting apoptosis through PI3K/Akt signaling^14^ and improve mitochondrial activity^15^. However, whether Rg1 exerts anti-apoptosis apoptotic effects in AA by affecting mitochondrial pathway function remains unknown.

The purpose of this study was to investigate whether Rg1 can be used to effectively treat the hematopoietic stem cells (HSCs) of AA by suppressing the mitochondrial apoptosis induced by Bax translocation.

## Methods

### Materials

Rg1, the molecular formula of which is shown in Fig. 2A, was purchased from Shanghai Woka Biotechnology Development (> 98% purity; Shanghai, China). FITC-labeled antibodies against lineage markers, including macrophage-1 antigen (Mac-1), Gr-1, Ter119, CD4, CD8a, CD3, B220, c-Kit-APC and Sca-1-PE, were purchased from BD Biosciences (Shanghai, China). A mitochondria isolation kit was purchased from Beyotime Biotechnology Inc. (Beijing, China). ROS detection kits were purchased from Solibao Technology Co., Ltd. (Beijing, China). A functional mitochondria isolation kit, mitochondrial outer membrane integrity testing kit, mitochondrial inner membrane integrity testing kit, purified mitochondrial cytochrome C oxidase activity assay kit, and cell monoamine oxidase (MAO) total activity colorimetry assay kit were obtained from GenMed Scientifics Inc. (Powell, OH, USA). Phosphate-buffered saline (PBS) was obtained from Wuhan Boster Biotechnology, Ltd. (Wuhan, China). The cell lysis buffer and horseradish peroxidase‑conjugated secondary antibodies used for Western blotting were obtained from Beyotime Biotechnology (Shanghai, China). Bcl-2, Bax, Bak, cleaved caspase-3,AIF, Apaf-1, Cyt-C, and β-actin were purchased from Abcam Co. (Cambridge, MA, USA).

### Patients and experimental protocol

Between September 2016 and February 2017, 8 patients (median age 43.6 years; range 32–64) were enrolled in this study. Eligible patients had a histologically confirmed diagnosis of AA. This study was approved by the Institutional Review Board of the Affiliated Hospital of Shandong University of Traditional Chinese Medicine, and written informed consent was obtained from all participants in accordance with the Declaration of Helsinki. Bone marrow mononuclear cells were obtained from patients for analyses of apoptosis, mitochondrial number, and Bcl-2/Bax mRNA level.

### Animals and experimental protocol

Sixty healthy BALB/c male mice weighing 18–22 g and aged 6–8 weeks were provided by the Experimental Animal Center of Shandong University (China). The animals were housed in a warm, quiet environment with free access to food and water and acclimatized for one week before the experiments began. All animal procedures were performed with the approval of the Animal Ethics Committee of the Affiliated Hospital of Shandong University of Traditional Chinese Medicine. All experiments were performed in accordance with relevant guidelines and regulations.

The 60 mice were randomly divided into several groups: a normal control group, the model group and three treatment groups. The AA model was established as previously described^1,6^. Briefly, the mice were irradiated with 5.0 Gy Co60 γ-radiation, and 2 × 10^6^ lymph node cells from DBA/2 donor mice were transplanted within 4 h after radiation.

The treatment groups were intraperitoneally injected with Rg1 (at 20, 40, and 80 mg/kg/day, for the low-dose group, medium-dose group and high-dose group, respectively, according to previous studies). The mice in the normal control and model groups were intraperitoneally injected with physiological saline (10 ml/kg/day). In addition, all the mice received a standard diet throughout the study. After treatment with Rg1 or physiological saline for 2 weeks, euthanasia was performed by cervical dislocation on day 14. Before euthanasia, blood was collected by puncturing the caudal vein, and after all of the animals were killed by cervical dislocation, the femur and spleen were removed immediately.

Blood samples were collected via the tail vein from the mice in all groups on day 14. Routine peripheral blood routine, counts of bone marrow mononuclear cells (BMNCs), burst-forming units-erythrocytes (BFU-E), and colony-forming units-erythrocytes (CFU-E) and bone marrow biopsy were conducted for verification of the model. Bax IHC was also performed. For the acute toxicity test, the maximum tolerated dose (500 mg/kg/day) of Rg1 was administered for 2 days. Bone marrow and liver data are shown in Supplementary Fig. 1, which showed no toxicity of Rg1 in mice.

### Cell sorting and culture for the in vivo and in vitro experiments with mice

Based on Nan’s study^17^, for the in vivo experiments, bone marrow cells were obtained from the groups of animals on day 14. Lin–Sca-1 + c-Kit + (LSK) populations were sorted from bone marrow cells without RBCs by using a BD FACSAria II flow cytometer. We used FITC-labeled antibodies against lineage markers, including Mac-1, Gr-1, Ter119, CD4, CD8a, CD3, and B220 (BD Biosciences), and anti-c-Kit-APC and anti-Sca-1-PE antibodies. Sorted cells were immediately analyzed tested in IMDM (Thermo Fisher Scientific).

For the in vitro experiment, bone marrow cells were obtained from all the groups of animals on day 7. After the LSK populations were sorted, LSK cells from the control and model groups were cultured in IMDMsupplemented with 10% FBS (Biological Industries, Beit-Haemek, Israel) and antibiotics (100 u/mL penicillin and streptomycin). LSK cells from the treatment groups were cultured in the same medium supplemented with FBS and antibiotics but also containing Rg1 (12.5, 25, or 50 μmol/L). These cells were cultured in a humidified incubator containing 5% CO_2_ at 37 °C and then analyzed.

### Analysis of apoptosis

Cell apoptosis of BMNCs from humans or LSKs from mice cultured for 48 h was quantified using an Annexin V-FITC kit (BD Biosciences, San Jose, CA, USA) according to the manufacturer’s instructions and analyzed by flow cytometry.

### Mitochondrial number assay of human BMNCs

Mitochondrial number in human BMNC cells was measured by using MitoTracker Green FM (Molecular Probes) as described by Mancini^18^. Cells were collected by trypsinization, suspended in PBS and fixed with a fixative containing 2% glutaraldehyde and 2% formaldehyde in PBS for MitoTracker Green FM staining. After being washed and suspended in PBS, the cells (3 × 10^5^ cells/mL) were stained with 75 nM MitoTracker Green FM for 30 min at room temperature in the dark, and then subjected to flow cytometric analysis.

### Measurement of mRNA

Genomic DNA for the analysis of mtDNA content was isolated from human BMNC cells or mouse LSK cells using a TaKaRa MiniBEST Universal Genomic DNA Extraction kit (TaKaRa Bio Inc.). The relative mtDNA copy number was determined by qPCR with primers for the mitochondrial 16S rRNA gene and the nuclear Actin gene as previously described^19^. All PCRs were performed in triplicate. The primers used to amplify 16S rRNA were 5′-GGTGCAGCCGCTATTAAAGG-3′ (16S rRNA, forward) and 5′-ATCATTTACGGGGGAAGGCG-3′ (16S rRNA, reverse).

For the measurement of Bcl-2 and Bax mRNA levels, the primers used were 5′-GACTGAGTACCTGAACCGGCATC-3′(Bcl-2, forward), 5′-CTGAGCAGCGTCTTCAGAGACA-3′(Bcl-2, reverse), 5′-ATGCGTCCACCAAGAAGC -3′(BAX, forward), and 5′-CAGTTGAAGTTGCCATCAGC-3′(BAX, reverse), according to a previous study^20^. The relative mRNA levels of Bcl-2 and Bax were normalized to ACTB, the primers used for ACTB were 5′-TGACGTGGACATCCGCAAAG-3′ (ACTB, forward); and 5′-CTGGAAGGTGGACAGCGAGG-3′ (ACTB, reverse).

### ROS staining

Cells were collected at the selected time point, the medium was discarded, and the cells were washed three times with 1 mL of PBS buffer. One milliliter of the fluorescent probe DCFH-DA was diluted to 1:1000 added to the cells, which were then incubated at 37 °C for 30 min. The cells were then washed three times with 1 mL of PBS buffer, counterstained with DAPI, and washed another 3 times. A confocal laser scanning microscope was used to image the cells and determine the distribution and expression of green fluorescence [indicative of reactive oxygen species (ROS)].

### Mitochondrial analyses by TEM morphometry

Mitochondrial structure was analyzed by electron microscopy. Then, images were obtained using a digital video camera (JVC, ky-F30B3-CCD, Japan), and the images were transferred to a computer. Then, the number, perimeter and area of each mitochondrial cross-section were calculated (Shen and Shen 1991). The measurements from all images were based on the same reference area and obtained by the image analysis system (Kontron Ibas 2.0 Germany). The quantitative density parameters [volume density (Vv) and numerical density (Nv)] and average surface area (S) were calculated based on the method of Ref.^21^.

### Mitochondrial lysis time curves in vitro

After mitochondria were extracted from LSK cells incubated in medium with or without Rg1, MAO and COX concentrations were detected as biomarkers of mitochondrial membrane lysis. The concentration of MAO indicates the concentration of mitochondria and was determined using 200 U/mL mitochondrial suspensions from serum cultures within 12 h of harvest at different time points (separated by 1-hintervals). The concentration of COX in the medium was also determined. The peak time analysis of the mitochondrial membrane and matrix-specific enzyme concentration–time curves revealed the trend in mitochondrial membrane lysis after treatment with Rg1.

### Determination of ATP, ADP and AMP concentrations in vitro

Perchloric acid and high-performance liquid chromatography (HPLC) were used to determine the ATP, ADP and AMP levels^22^. LSK cells were lysed in 50% perchloric acid and then centrifuged (at 4 °C and 12,000 rpm for 30 min). As soon as the supernatant was transferred to a new centrifuge tube, 2.5 M K_2_CO_3_ was added, and the solution was centrifuged under the same conditions. Then, the supernatant was immediately analyzed by HPLC under the following detection conditions: HYPERSIL C18 5u analytical column; 250 mm column length; 4.6 mm column diameter; 0.005 mol/L H_2_PO_4_ (pH 6.0) mobile phase, and UV 254 nm (H_2_PO_4_) detection. The results are expressed as µg/g protein.

### Western blot analysis

LSK cells obtained from mice on day 14 or cultured for 48 h in vitro were lysed with cell lysis buffer for Western blotting. Forty micrograms of protein per sample was separated on a 12% SDS‑PAGE gel. The proteins were then transferred electrophoretically to 0.45 μm nitrocellulose membranes, which were then incubated overnight with primary antibodies against Bcl‑2, Bax, Bak, cleaved caspase 3, Cyt-C, Apaf-1, AIF and β-actin at 4 °C. Next, the membranes were incubated with secondary antibodies. The band intensities were analyzed by ImageJ software (ImageJ version 1.47, National Institutes of Health, Bethesda, MD; available at: https://imagej.nih.gov/ij/).

### Colocalization analysis of mitochondria with Bax in vitro

The colocalization of mitochondria with Bax was observed by confocal fluorescence microscopy^23^. To explore the connection between mitochondria and Bax, LSK cells were cultivated on cell slides for staining. For labeling the mitochondria, MitoTracker Red CMXRos (Thermo Fisher Scientific, M7512) (500 nM) was added to living cells at 37 °C for 30 min and then fixed with 4% PFA. Then, 0.1% Triton X-100 was used to permeate the cell membrane for 5 min at room temperature. The cell slides were incubated with Bax antibodies (1:100) at 4 °C overnight. After incubation with secondary antibody, the slides were analyzed by fluorescence microscopy at 550 nm for MitoTracker Red CMXRos and the respective wavelengths for the fluorescence secondary antibodies.

### Statistical analysis

Statistical analysis was performed using a computer software SPSS Version19.0 (SPSS Inc., Chicago, IL, USA). The data are expressed as the means ± SDs. One-way ANOVA was used to test the significance of differences among groups, followed by Scheffe’s modified F-test for multiple comparisons. A value of P < 0.05 was considered statistically significant.

### Ethics approval and consent to participate

All experiments were conducted in compliance with the ARRIVE guidelines. We confirm that animal care and experimental procedures were carried out in accordance with the guidelines of the Animal Ethics Committee of the Affiliated Hospital of Shandong University of Traditional Chinese Medicine. The reference number is 2015-5-07.

## Results

### The mitochondrial apoptosis in BMNCs

**Figure 1:**
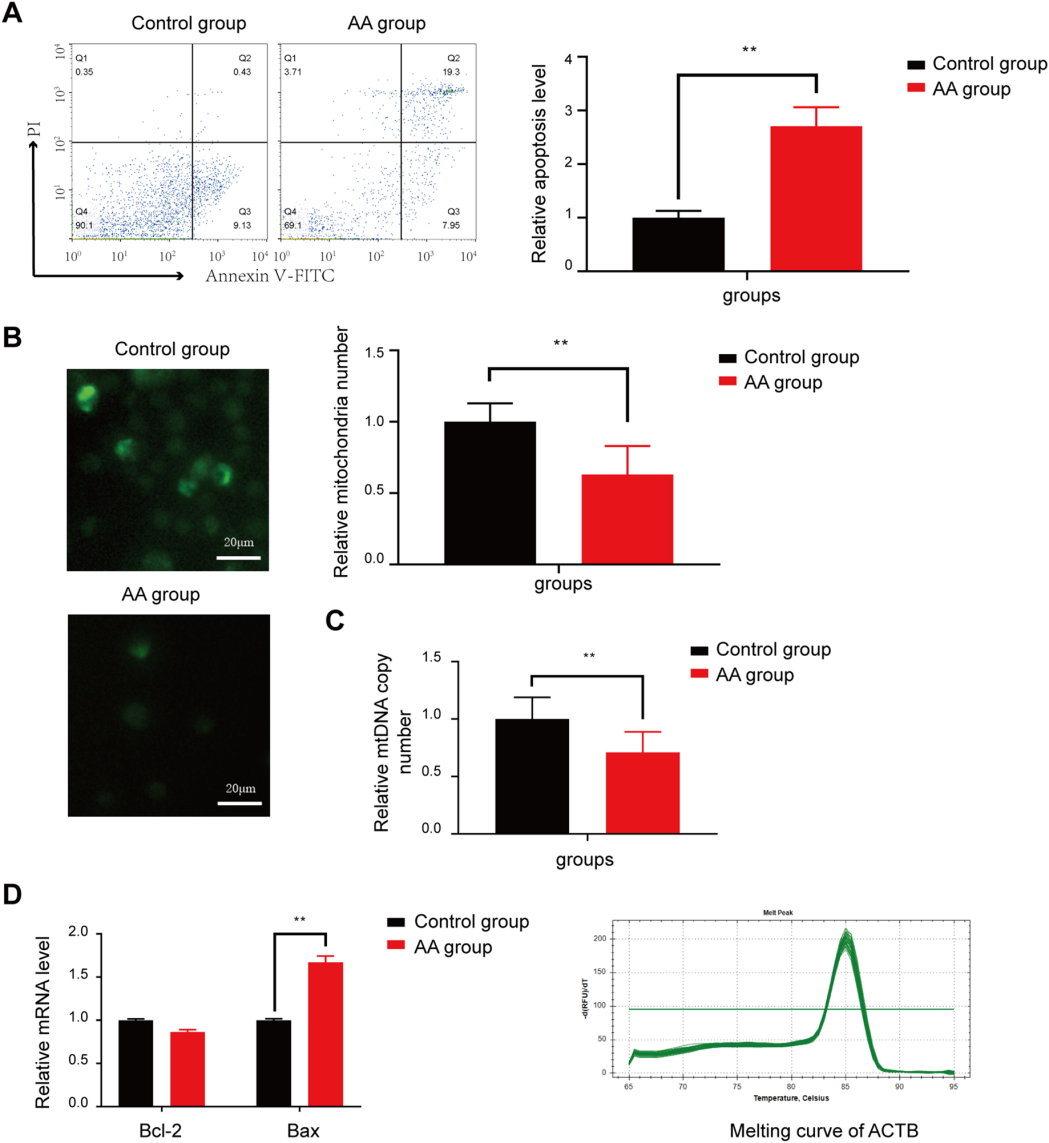
Assessment of mitochondrial apoptosis in AA patients. (**A**) Apoptosis level in AA patients and normal controls. (**B**) Mitochondrial number determined using molecular probes. (**C**) Mitochondrial DNA copy number determined by real-time PCR. (**D**) Bcl-2 and Bax mRNA levels determined by real-time PCR. **P < 0.01 compared with the control group.

There were significantly higher levels of apoptosis in BMNCs from AA patients than in those from normal individuals according to the flow cytometry data (Fig. 1A). As presented in Fig. 1B, the mitochondrial number determined by using molecular probes was significantly lower in the BMNCs from AA patients’ than in those from normal individuals (P < 0.01). We also analyzed the number of mitochondrial DNA copies by real-time PCR. There was greater loss of mtDNA in AA patients than in control subjects (Fig. 1C). To verify this result, we investigated the levels of mitochondrial apoptosis-associated markers. The relative level of Bax of AA patients was significantly increased compared with that of normal individuals, whereas no marked decrease in Bcl-2 levels were observed in AA patients (Fig. 1D).

### Effect of Rg1 treatment on the recovery of hemopoietic function in AA mice

**Figure 2:**
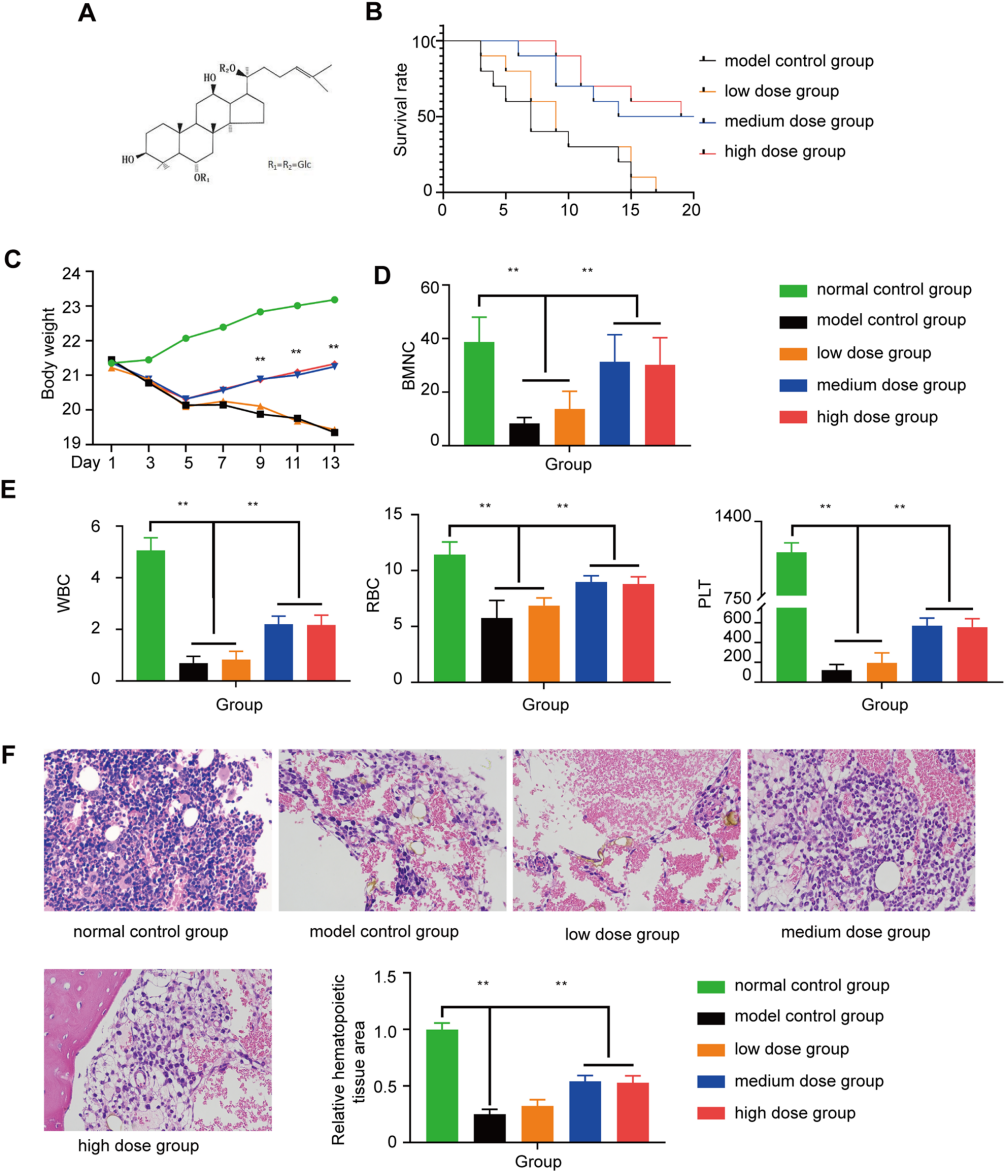
Assessment of the effect of Rg1 on the AA mouse model. (**A**) The structural formula of Rg1. (**B**) Survival rates of mice treated with different Rg1 doses. (**C**) BMNC counts (x ± SD) in the different groups. (**D**) Peripheral blood cell counts (x ± SD) in the different groups. (**E**) Bone marrow biopsy of the normal group, model control group and different dose Rg1 treatment groups on day 14. The hematopoietic areas in the treatment groups were appreciably larger than the area in the AA group. *P < 0.05 and **P < 0.01 compared with the model control group.

Using a fully automated blood cell analyzer, we found that the numbers of peripheral blood cells and BMNCs and the area of hematopoietic tissue in bone marrow biopsies from the AA mice were notably decreased compared to those from the control mice (P < 0.01), indicating the successful establishment of the mouse model of AA (Fig. 2). Treatment of these mice with medium- or high-dose Rg1 for 14 h increased survival time (P < 0.01; Fig. 2B), and WBC, RBC, PLT and BMNC counts as well as weight in the treated mice were increased compared with those of the model control group (P < 0.01; Fig. 2C–E).

### Effect of Rg1 treatment on BFU-E and CFU-E in AA mice

**Figure 3:**
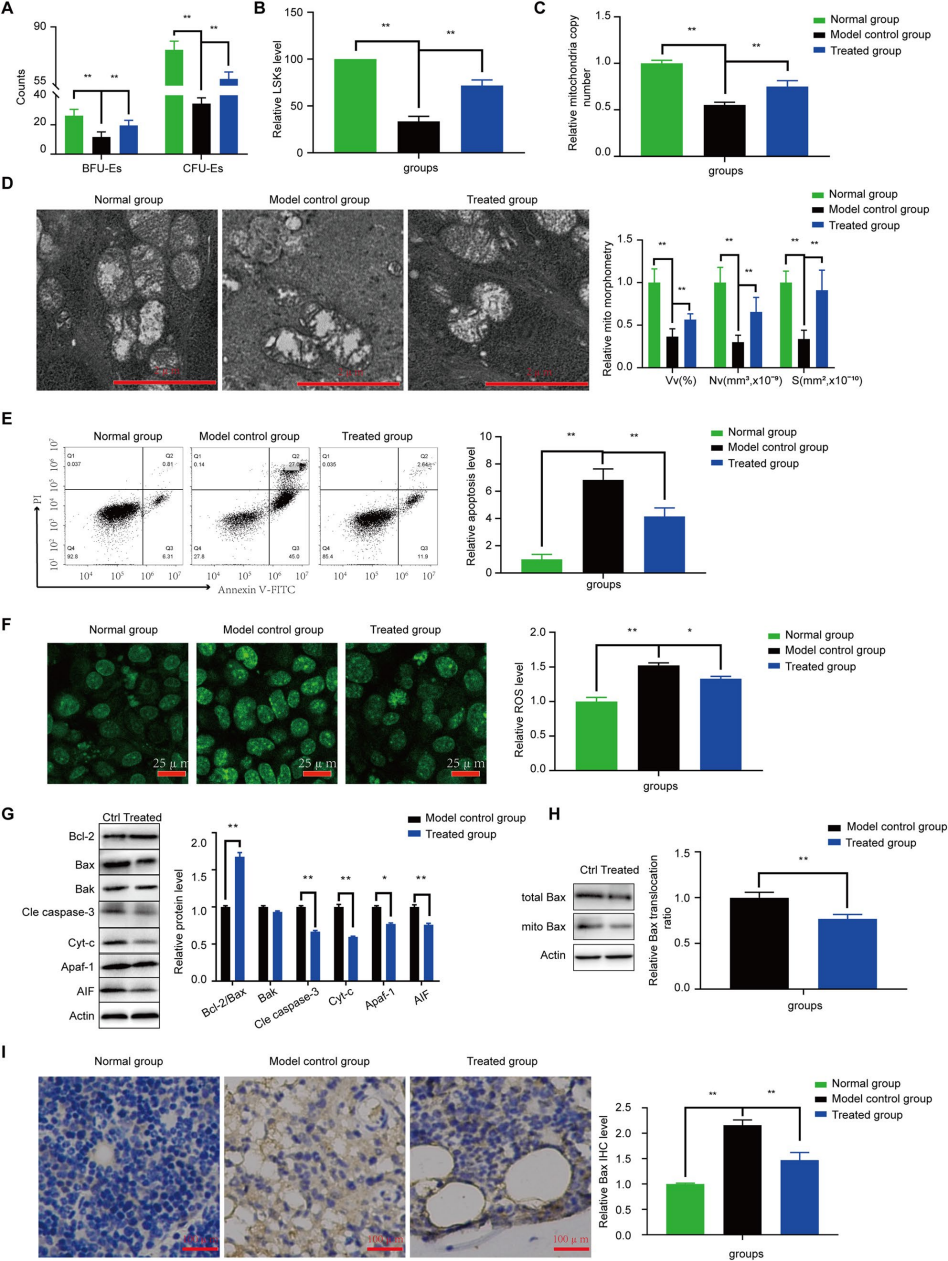
Effect of Rg1 on the recovery of hematopoietic function, mediated by the alleviation of Bax translocation-induced mitochondrial apoptosis, in LSK cells obtained from mice on day 14. (**A**) The numbers of BFU-Es and CFU-Es in different AA groups was detected to assess the efficacy of medium-dose Rg1. (**B**) The number of LSK cells in the control, model AA, and Rg1 treatment groups. (**C**) Mitochondrial DNA copy number determined by using real-time PCR. (**D**) Mitochondrial structure was detected by electron microscopy on day 14. (**E**) Apoptosis levels in LSKs determined by using flow cytometry. (**F**) Ros levels in LSK cells obtained from mice. (**G**) Levels of mitochondrial apoptosis-related proteins in LSKs. (**H**) The Bax ratio of LSKs in mitochondria and total. (**I**) Bax immunohistochemistry in different groups. *P < 0.05 and **P < 0.01 compared with the model control group.

The counts of BFU-E and CFU-E in the bone marrow of AA mice were found to be significantly lower than those in the bone marrow of normal control mice (P < 0.01). Furthermore, microscopy revealed depressed growth and proliferation of erythroid progenitor cells in the AA mice. After treatment with medium-dose Rg1 (40 mg/kg/day), the BFU-E and CFU-E counts were restored to 66.8% (17.5/26.2) and 77.25% (59.1/76.5), respectively, of the normal levels (Fig. 3A).

### Effect of Rg1 treatment on mitochondrial quantity of LSKs

**Figure 4:**
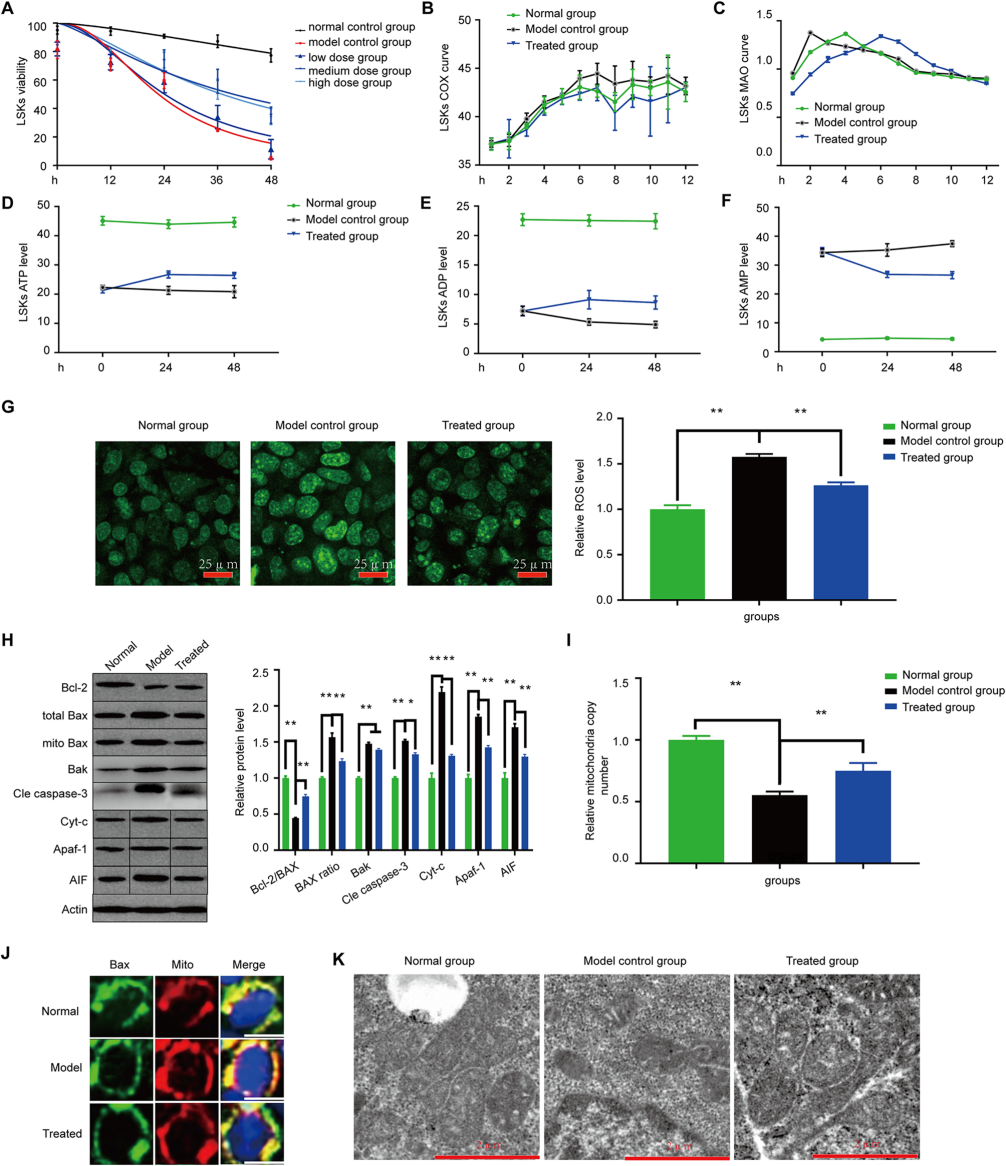
Effect of Rg1 on mitochondrial apoptosis of LSK cells in vitro. (**A**) The cell viability of LSK cells was detected by PrestoBlue Assay. (**B**) and (**C**) As markers of kinase activity in the outer membrane and inner membrane, respectively, MAO and COX levels were determined in LSK cells, and concentration–time curves of MAO/COX were constructed to determine the efficacy of Rg1. (**D**–**F**) The ATP/ADP/AMP levels in different groups. (**G**) Ros levels in different groups. (**H**) The protein test of Bax translocation-induced mitochondria. (**I**) Mitochondrial DNA copy number determined by real-time PCR. (**J**) The colocalization of Bax with mitochondria (yellow dots). (**K**) Mitochondrial structure was detected by electron microscopy. *P < 0.05 and **P < 0.01 compared with the model control group.

After treatment with 40 mg/kg/day Rg1 or saline solution for 14 days, LSK cells were obtained from mouse bone marrow for mitochondrial apoptosis tests. LSK level significantly recovered in the treatment group (Fig. 3B), and the data were similar to the in vitro data (Fig. 4A). Rg1 also increased mitochondrial number (Fig. 3C) and alleviated the apoptosis level (Fig. 3D) compared with the number and level observed in the model group (P < 0.01). Transmission electron microscopy of the mouse bone marrow showed that the size and shape of the mitochondria were normal in the control group. In contrast, the mitochondria in the model control group exhibited enlarged globular structures and the disruption or disappearance of cristae (Fig. 3D). Mitochondrial number was significantly improved after treatment with Rg1, indicating that Rg1 recovered mitochondrial number in AA mice.

### Effect of Rg1 treatment on mitochondrial apoptosis in LSKs

The levels of apoptosis and related proteins were investigated in LSKs. The flow cytometry data showed that Rg1 could alleviate the abnormal increased in apoptosis observed in the model control group (Fig. 3E). The mechanism was as follows: Rg1 decreased the levels of mitochondrial apoptosis markers such as Bax, Cyt-C, Apaf-1 and AIF and cleaved caspase 3(Fig. 3G), by inhibiting the production of ROS (Fig. 3F). Importantly, there was no significant decrease in Bak level after Rg1 treatment(Fig. 3G). The Bax translocation ratio is important in the induction of mitochondrial apoptosis, and Rg1 can inhibit this induction (Fig. 3H). The Bax IHC data supported these results (Fig. 3I).

### Effect of Rg1 treatment on the mitochondrial lysis time curve

In the control group, the COX level peaked after 3.5 h. The MAO level in the culture did not change significantly over time, but the COX level gradually increased with time until peaking and then gradually declining. Thus, the complete cleavage of mitochondrial contents released by cells required approximately 3.5 h to peak.

The COX peaks in the AA group appeared at 1.5 h and 1.375 U/L. The COX peaks in the treatment group appeared at 5.5 h and 1.341 U/L. At 1.5 h, the COX level in the AA group was significantly higher than that in the treatment group (P < 0.05).

Therefore, the complete cleavage of mitochondrial contents in the serum was significantly delayed after the addition of Rg1 (P < 0.05), and the peak was slightly lower than that in the AA group (Fig. 4B,C).

### Influence of Rg1 treatment on the recovery of energy generation in vitro

The ATP/ADP level indicates the ability of HSCs to generate energy. The ATP/ADP level in the AA group and treatment group decreased gradually over time, but was higher in the treatment group than in the AA group at 24 h and 48 h (P < 0.05; Fig. 4D,E). In contrast to the changes observed in ATP level, the AMP level in model and treatment groups was significantly elevated relative to the control level and increased markedly in the AA group. At 48 h, the AMP content in the treatment group was lower than that in the AA group (P < 0.05; Fig. 4F). These data showed that Rg1 could ameliorate the dysregulation of energy generation in AA.

### Influence of Rg1 treatment on Bax translocation-related mitochondrial apoptosis

The levels of protein markers of mitochondrial apoptosis were detected to determine the effects of Rg1 in vitro. The quantitative protein expression data were normalized to β-actin expression and are shown as a percentage of the expression in the control group and as the means ± standard deviations (n = 3). The data demonstrated that Rg1treatment decreased the ROS level (Fig. 4G) and rectified the abnormal protein levels of Bcl-2/Bax by decreasing the levels of Bax, Cyt-C, Apaf-1, AIF and cleaved caspase 3 at 48 h (P < 0.01) (Fig. 4H). However, these levels were not restored to normal levels either in vivo or in vitro (P < 0.01). The Bax translocation level was then assessed by using Western blotting, and mitochondrial colocalization with Bax was investigated. Rg1 decreased the ratio of Bax mitochondrial translocation (Fig. 4H) in vivo and alleviated the colocalization of Bax with mitochondria (Fig. 4J). Rg1 also restored mitochondrial number and structure, as revealed by PCR and TEM analyses (Fig. 4I,K).

## Discussion

Mitochondrial dysfunction and a decrease in mitochondrial number may result in a reduction in mtDNA. As evidence of acquired mtDNA deletions in the hematopoietic compartment has been found, mtDNA mutations and severe pancytopenia or reticulocytopenia are believed to be closely related^24^. Events such as mtDNA damage and abnormal mtRNA transcription, protein synthesis, and mitochondrial function can lead to mitochondrial injury, and both mtDNA mutation and mtDNA copy number reduction are causes of disease^25,26^. If the damaged mitochondria are cleared, erythrocyte maturation and homeostasis can be accelerated^27^. Studies have clarified that mitochondrial apoptosis is a key factor in AA. ROS-dependent pathway is always an important pathway which leads to the apoptosis death via mediating Bax translocation^28^ or JNK-p38^29^ and other pathways. Increased ROS defects contribute to severe combined anemia and thrombocytopenia^30^. By inhibiting abnormal mitochondrial oxidative phosphorylation, rapamycin can ameliorate the phenotype of the immune-mediated AA model^31^. If mitochondrial dysfunction is decreased, the genomic and functional integrity in the hematopoietic system can be protected^32^. Furthermore, in the AA rat model, the reversal of abnormal levels of mitochondrial DNA content and function, can restore the characteristics of healthy rats^33^.

Rg1 has been used treat anemia and bone marrow damage for many years. It can alleviate hematopoietic homeostasis defects^34^, delay hematopoietic stem/progenitor cell senescence by influencing the SIRT6/NF-κB signaling axis^35^, and protect against HSC aging by regulating the SIRT1-FOXO3 and SIRT3-SOD2 signaling pathways^36^ or by regulating bone marrow stromal cells^37^ to recover hematopoietic function. The anti-apoptosis, effects of Rg1 have been proven in several studies, with Rg1 being capable of protecting cardiomyocytes^38^ and lung epithelial cells^39^.In addition, in hematology studie, Rg1 has been shown to inhibit mitochondrial dysfunction^40^ and, delay senescence in BMNCs^37^. Rg1 can also inhibit apoptosis by decreasing Bax level and restore an abnormal Bcl-2/Bax ratio to normal levels^36,41,42^. Therefore, we investigated whether Rg1 can mitigate AA by preventing mitochondrial apoptosis in hematopoietic cells.

In our study, significant apoptosis and a decrease in mitochondrial number were found in AA patients due to an abnormal Bcl-2/Bax ratio (Fig. 1). We established a mouse model of AA by applying a combination of 60Co γ-radiation and transplantation with lymph node cells from DBA/2 donor mice. The AA mice showed statistically significant reductions in the measures of peripheral blood leukocytes, hemoglobin and PLTs and severe reductions in the numbers of BMNCs (Fig. 2B–D) and marrow-committed progenitor cells (Fig. 3A), which are clinical characteristics of AA. Furthermore, unbalanced Bcl-2/Bax ratio-induced mitochondrial apoptosis was verified in the mouse model (Fig. 3G,I). AA mice treated with Rg1 showed an increase in the number of BMNCs (Fig. 2C), and with the increases in BFU-E and CFU-E counts (Fig. 3A), LSK cell levels increased (Figs. 3B, 4A) compared with those in the AA group. Additionally, the increase in mitochondrial number (Figs. 3C, 4I) and the decreases in mitochondrial apoptosis level in vivo and in vitro verified the positive effect of Rg1 in AA mice (Figs. 3E–I, 4H). Similar to Meng’s data^43^, Rg1 is proved to decrease the ROS level (Fig. 3F, 4G) which is a main reason for preventing apoptosis. Moreover, the anti-apoptosis effect of Rg1 was found to be mediated by its inhibition of Bax translocation, as evidenced by the decrease in the level of colocalization of Bax with mitochondria following Rg1 without a significant decrease in Bak level. Furthermore, we found that Rg1 treatment corrected the abnormal mitochondrial lysis time curve and energy level of LSK cells in vitro (Fig. 4B–F). To further explore the mechanism, we analyzed the Bax ratio data of mitochondria and total and found that Rg1 inhibited Bax assembly from the cytoplasm in mitochondria (Figs. 3H, 4H). The colocalization of Bax with mitochondria was inhibited by Rg1 (Fig. 4J), resulting in apoptosis resistance (Fig. 3E). These results showed that Rg1 recovered hematopoietic function by promoting BMNC proliferation, increasing mitochondrial number, stabilizing the mitochondrial membrane and restoring the energy supply by inhibiting Bax translocation-induced mitochondrial apoptosis, and that Rg1 treatment prolonged the survival of AA mice.

In brief, AA model mice exhibit severe issues with mitochondrial apoptosis that can be ameliorated by treatment with Rg1. Thus, therapeutic targets that can maintain mtDNA integrity and copy number may be considered crucial players in the treatment of AA.

## Supplementary Information

Supplementary Figure 1.

## Data Availability

The datasets supporting the conclusions of this article are included within the article. The datasets used and/or analyzed during the current study are available from the corresponding author on reasonable request.

## Abbreviations

Rg1: Ginsenoside Rg1
AA: Aplastic anemia
BMNCs: Bone marrow mononuclear cells
MDA: Malondialdehyde
COX: Cytochrome oxidase
mtDNA: Mitochondrial DNA
CFU-Es: Colony-forming units-erythrocytes
BFU-Es: Burst-forming units-erythrocytes
