# Supplementary material for: Ginsenoside Rg1 can restore hematopoietic function by inhibiting Bax translocation-mediated mitochondrial apoptosis in aplastic anemia
Source: Sci Rep. 2021 Jun 17;11:12742. doi: 10.1038/s41598-021-91471-1 (PMC8211841; doi:10.1038/s41598-021-91471-1)

***Supplementary materials***

***Ginsenoside Rg1 can restore hematopoietic function by inhibiting Bax translocation-mediated mitochondrial apoptosis in aplastic anemia***

Huiqin Cao^1&^, Wei Wei^2&^, Ruirong Xu^3^, Xing Cui^3*^

1 Department of Hematology, Affiliated Hospital of Yan 'an University, Yanan 716000, China;

2 Department of Neurosurgery, Affiliated Hospital of Yan 'an University, Yanan 716000, China;

3 Department of Hematology, Affiliated Hospital of Shandong University of Traditional Chinese Medicine, Jinan 250014, China;

^&^ *These authors contributed equally to this work and should be considered as co-first authors.*

**Running title:** Correct mitochondrial apoptosis in aplastic anemia

^*^**Corresponding author:**

Xing Cui

Department of Hematology

Affiliated Hospital of Shandong University of Traditional Chinese Medicine

16369 Jingshi Road

Jinan, Shandong Province, 250014, China

Tel:86+68616042

Fax: 86+68616042

E-mail:cdz45@foxmail.com.

**Figure legends**

**Fig. 1.** Representative histopathological sections of the (A) bone marrow and (B) liver of mice treated with 500 mg/kg/d of Rg1 for 2d. The control group was not treated. Insignificant lesions were observed in both tests. Hematoxylin-eosin stain, 200× original magnification.


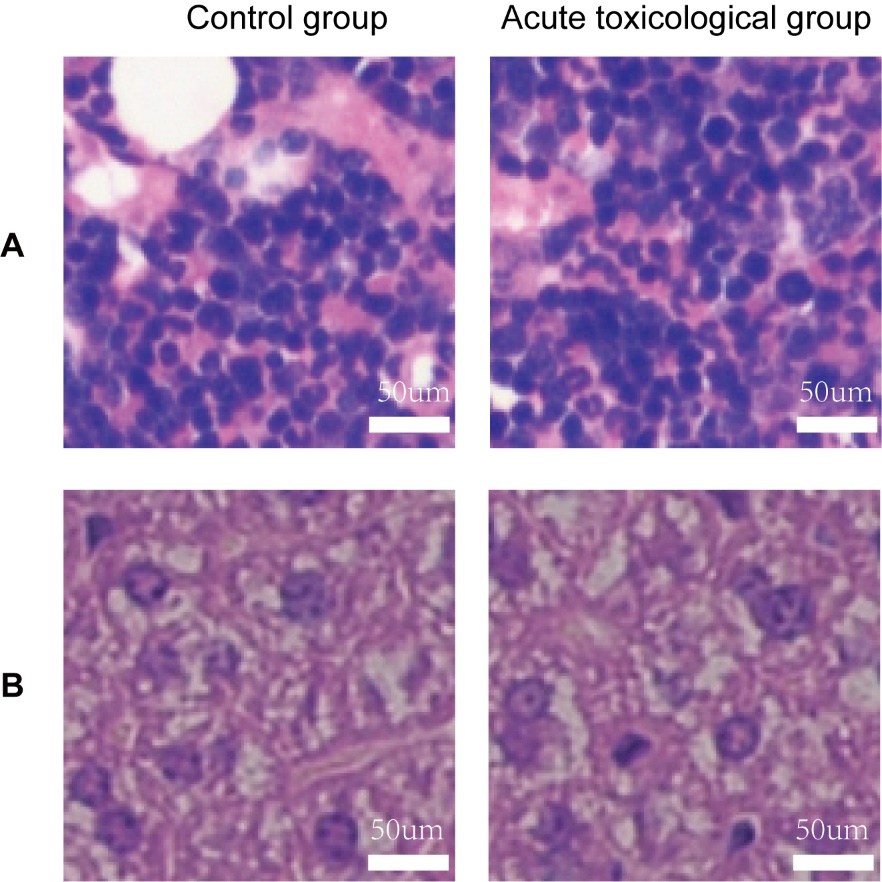


**Fig.**

The original Bcl-2 gel image ( Fig 3G )


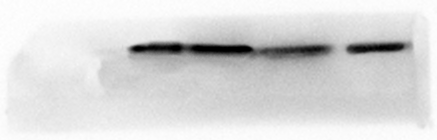


The original Bcl-2 gel image ( Fig 4H)


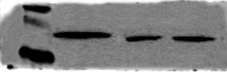


The original Bak gel image ( Fig 3G and Fig 4H)





The original BAX gel image ( Fig 3 G and H)


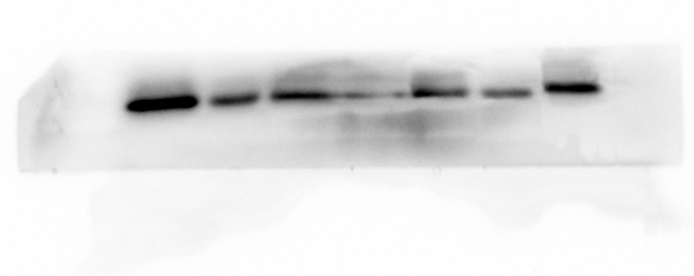


The original BAX gel image ( Fig 4H)


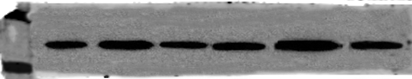


The original cle caspase-3 gel image ( Fig 3G and Fig 4H)


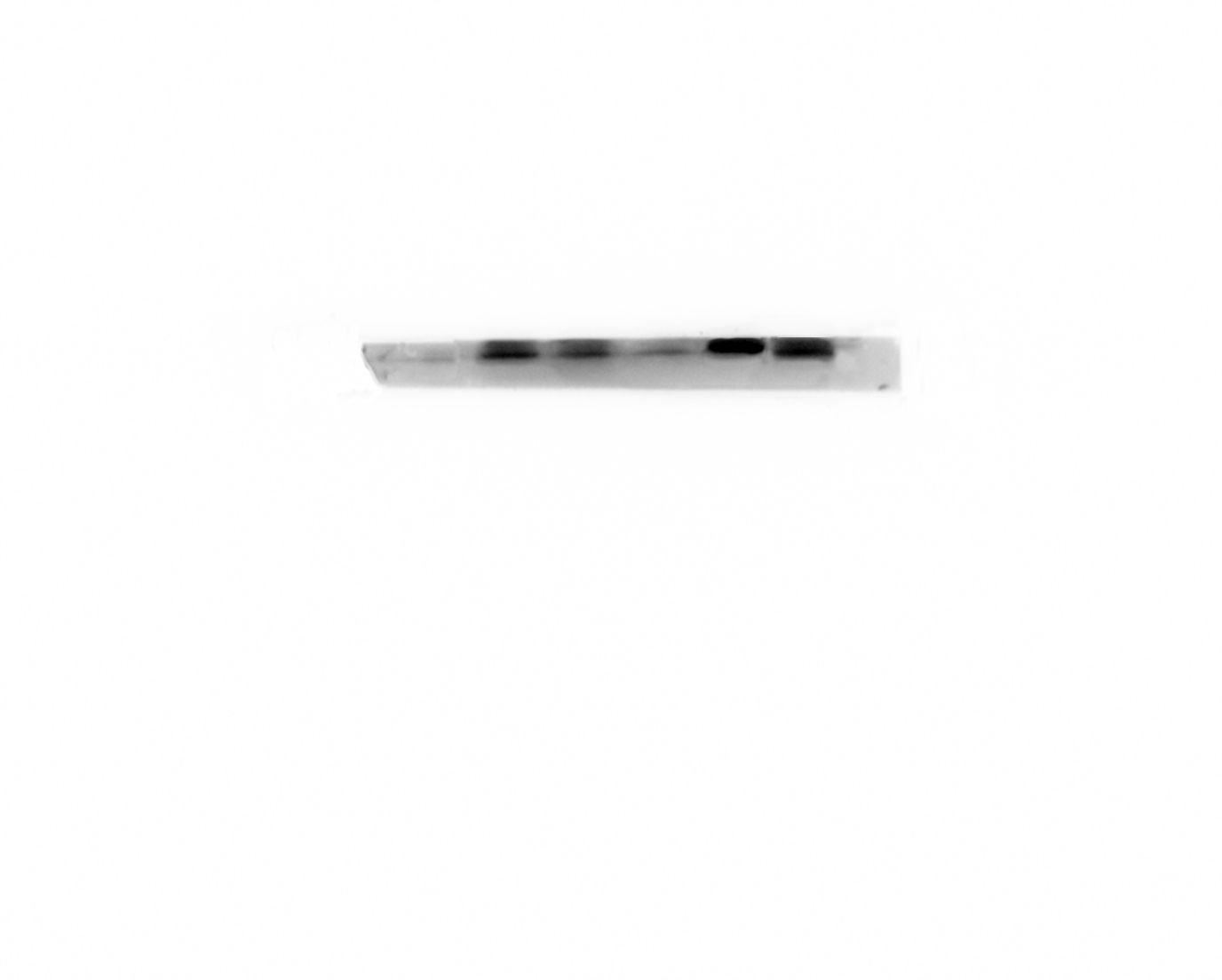


The original Cyt-c gel image ( Fig 3G )


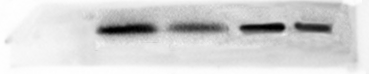


The original Cyt-c gel image ( Fig 4H )


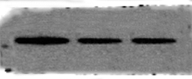


The original Apaf-1 gel image ( Fig 3G )


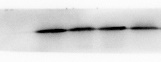


The original Apaf-1 gel image ( Fig 4H )


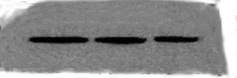


The original AIF gel image ( Fig 3G )


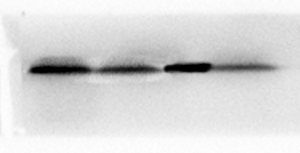


The original AIF gel image ( Fig 4H )


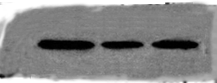

Supplement: Supplementary file 1 — Supplementary Figure 1. [file 41598_2021_91471_MOESM1_ESM.docx]
